# Angle Surgery in Pediatric Glaucoma Following Cataract Surgery

**DOI:** 10.3390/vision5010009

**Published:** 2021-02-05

**Authors:** Emery C. Jamerson, Omar Solyman, Magdi S. Yacoub, Mokhtar Mohamed Ibrahim Abushanab, Abdelrahman M. Elhusseiny

**Affiliations:** 1 Department of Ophthalmology, Columbia University Irving Medical Center, Edward S. Harkness Eye Institute, New York, NY 10032, USA; ecj2121@cumc.columbia.edu; 2 Department of Ophthalmology, Research Institute of Ophthalmology, Cairo 11261, Egypt; o.solyman2@gmail.com (O.S.); mokh211@yahoo.com (M.M.I.A.); 3 Department of Ophthalmology, Kasr Al-Ainy Hospitals, Cairo University, Cairo 11261, Egypt; magdysyacoub@gmail.com; 4 Department of Ophthalmology, Boston Children’s Hospital, Harvard Medical School, Boston, MA 02115, USA

**Keywords:** trabeculotomy, goniotomy, glaucoma following cataract surgery (GFCS), intra-ocular pressure (IOP), angle surgery, microcatheter

## Abstract

Glaucoma is a common and sight-threatening complication of pediatric cataract surgery Reported incidence varies due to variability in study designs and length of follow-up. Consistent and replicable risk factors for developing glaucoma following cataract surgery (GFCS) are early age at the time of surgery, microcornea, and additional surgical interventions. The exact mechanism for GFCS has yet to be completely elucidated. While medical therapy is the first line for treatment of GFCS, many eyes require surgical intervention, with various surgical modalities each posing a unique host of risks and benefits. Angle surgical techniques include goniotomy and trabeculotomy, with trabeculotomy demonstrating increased success over goniotomy as an initial procedure in pediatric eyes with GFCS given the success demonstrated throughout the literature in reducing IOP and number of IOP-lowering medications required post-operatively. The advent of microcatheter facilitated circumferential trabeculotomies lead to increased success compared to traditional <180° rigid probe trabeculotomy in GFCS. The advent of two-site rigid-probe trabeculotomy indicated that similar results could be attained without the use of the more expensive microcatheter system. Further studies of larger scale, with increased follow-up, and utilizing randomization would be beneficial in determining optimum surgical management of pediatric GFCS.

## 1. Introduction

Glaucoma is among the most common causes of vision loss after successful congenital cataract surgery [1]. The majority of glaucoma following cataract surgery (GFCS, previously termed aphakic and/or pseudophakic glaucoma) is of the open angle variety; cases of post-operative acute angle closure and pupillary block glaucoma have been documented in the literature, but they are extremely rare and were largely associated with various intra-operative factors [2]. While the mechanism for GFCS has yet to be completely elucidated, prior studies have implicated post-operative trabecular meshwork (TM) obstruction caused by both anterior repositioning of the iris and also residual lens material, as well as synechiae formation, reduction in the diameter of Schlemm’s canal, and anterior chamber angle narrowing [3,4,5,6]. These factors contribute to an increase in intra-ocular pressure (IOP) after cataract surgery, not uncommonly leading to ocular hypertension (OHTN) and, in some cases, glaucoma [7,8]. The incidence of such occurrences varies throughout the literature from 15% to 45%, likely due to differences in study design, variability in age at time of surgery, sample size, and length of post-operative follow-up [9,10,11]. Generally, studies with longer follow-up found increased rates of GFCS, highlighting its variable time course and the importance of continued screening [12,13]. The main factors associated with increased risk of GFCS worldwide include shorter axial length, microcornea, need for additional surgical interventions, and younger age at surgery, with various critical periods having been posited—1 month, 9 months, 1 year—before which cataract surgery comes with a dramatic increase in risk of glaucoma [14,15,16,17,18,19,20,21,22]. Time between cataract surgery and glaucoma diagnosis has ranged in the literature from 2.5 months to over 5 years [11,23,24]. In addition, associations between risk of GFCS and various factors of the cataract surgery have been investigated, such as aphakia vs. pseudophakia after cataract extraction, and use of intra-operative anti-inflammatory agent [25,26,27,28,29,30]. These factors, however, have failed to demonstrate a reproducible and significant association with increased risk of GFCS.

When recognized early and managed appropriately, children with GFCS have been shown to demonstrate good visual outcomes [31]. Topical pressure lowering therapy is first-line treatment for GFCS, consisting of beta-blockers, prostaglandin agonists, and/or systemic carbonic anhydrase inhibitors. Alpha-2 agonist (Brimonidine) use is avoided in children less than the age of 2 due to risk of central nervous system depression [32]. Echothiophate iodide has also been posited as a potential therapy in pediatric GFCS with successful lowering of IOP with minimal adverse outcomes [33]. In many patients, however, maximal medical therapy alone is insufficient to control IOP and halt disease progression, and surgical therapy is also required [34]. Options for surgical management of GFCS have historically included glaucoma drainage implants, trabeculectomy with and without antimetabolites, such as mitomycin C and 5-fluorouracil, and cyclophotocoagulation (CPC) in many difficult or refractory cases [35,36,37]. Trabeculectomy has historically poor success rates in GFCS, precludes contact lens use (which is of great importance in aphakic eyes), and begets bleb-related complications, such as infection, leak, and over filtration [38]. Glaucoma drainage implants have demonstrated superior success rates to trabeculectomy, but also beget greater complication rates, particularly of suprachoroidal hemorrhage in the setting of hypotony, as well as keratopathy due to implant-related endothelial compensation [39,40,41,42,43]. CPC can be difficult to titrate and causes greater inflammation and carries a greater risk of phtisis [35,36,37,44]. Angle surgery in GFCS was first described by Chen et al., which posited angle surgery as a potential successful method in management of GFCS addressing physiological outflow pathways with fewer complications than previously-described surgical options [12]. The focus of this review is to outline the current literature on surgical methods and outcomes in angle surgery for pediatric GFCS to aid in management of such an insidious and sight-threatening disease entity.

## 2. Historical Development of Angle Surgery Techniques

Angle surgery, including goniotomy and trabeculotomy, have been historically utilized as first-line procedures in pediatric glaucomatous eyes without additional ocular or systemic abnormalities and with corneal diameters less than 14 mm. Goniotomy, first utilized in 1893, is the oldest surgical treatment option for congenital glaucoma, utilizing sharp instruments through a clear corneal incision to incise the trabecular meshwork for an arc of 110–120° opposite the corneal incision site [45]. This procedure allows for direct visualization of TM, more targeted cutting of abnormal tissue, and may be subsequently repeated. Goniotomy demonstrated success in management of congenital glaucoma; initial reports detailed an 80% success rate, defined by adequate IOP control without topical IOP lowering therapy [46]. Trabeculotomy (ab externo) was first described in 1960, and involved dissection through an incision radial to the limbus to unroof Schlemm’s canal using a trabeculotome to allow increased aqueous humor drainage [47]. In 1966, the procedure was modified to use an external scleral flap similar to trabeculectomy, and the trabeculotome was modified to have two parallel arms, one to guide direction of the trabeculotome, and the other to open Schlemm’s canal (now known as the Harm’s trabeculotome) [48]. Both goniotomy and trabeculotomy require pre-operative or intracameral miotic agents to reduce risk of lens damage during the procedure. When compared to goniotomy, trabeculotomy was found to have similar success rates, without a clear consensus on one procedure being superior to the other in management of congenital glaucoma [49]. Goniotomy has, however, demonstrated decreased success in patients with cloudy corneas, as well as patients beyond the first three years of life, which represents a great segment of patients with GFCS [50]. Trabeculotomy may be the procedure of choice in such patient populations. The outcomes of angle surgery in congenital glaucoma have been thoroughly described in the literature, but there are few studies that discuss outcomes of these procedures specifically in GFCS.

## 3. Goniotomy vs. Trabeculotomy as Initial Procedure

Table 1 summarizes experimental design and participant parameters in studies investigating angle surgery in GFCS, and Table 2 summarizes pre- and post-operative results in these same studies. In Chen et al. (2004), it was noted that 24 out of 97 eyes with aphakic glaucoma received goniotomy or trabeculotomy with a success rate of 16.0%, as defined by IOP ≤ 21 mmHg with or without medications and no need for further surgery [12]. These results, however, were not stratified by goniotomy vs. trabeculotomy as initial procedure, and only 9 of these eyes had these angle surgery procedures alone. In addition, the degree of trabeculotomy and surgical method was not explicitly stated. In order to compare success rates between goniotomy and trabeculotomy in patients with GFCS, Bothun et al. (2010) conducted a retrospective case series consisting of 14 eyes with aphakic GFCS [51]. Patients with anterior segment dysgenesis, microcornea, and glaucoma at time of cataract surgery were excluded. Procedures were performed by 4 different surgeons between 1990 and 2006 in the US with 10 right eyes and 4 left eyes as operative eyes. Patients received a 180° goniotomy or trabeculotomy as initial surgery, with the lateral 180° manipulated on initial trabeculotomy (one patient, however, received 360° suture trabeculotomy, the rest received trabeculotomy with Harm’s trabectome). The procedure for each eye was chosen according to the different surgeons’ standard first choice at the time of the study. After the completion of each case, dexamethasone and cefazolin were given, and a combination antibiotic/steroid drop given for up to 1 month after the procedure, with all IOP-lowering drops discontinued post-operatively and reinitiated for IOP > 21 mmHg associated with increased optic disc cupping or axial length. Patients were aged 3 months to 9.5 years, with a median age of 3 years with initial surgery occurring at a median of 2.9 years after initial cataract surgery. Follow-up occurred for an average of 4.7 years after the procedure. Success was defined as IOP ≤ 24 mmHg with or without topical medication, lack of sight-threatening complication, and avoidance of trabeculectomy or tube shunt. 2 Eyes had goniotomy alone, 3 eyes had goniotomy followed by trabeculotomy and 9 eyes had trabeculotomy. Eight eyes achieved treatment success, with 6 eyes, achieving treatment success after one surgery (all of which received trabeculotomy first). IOP was lowered from an average of 35 mmHg preoperatively to 22 mmHg postoperatively (*p* = 0.0005). Three eyes needed subsequent shunt placement after the last angle surgery because of progression of glaucoma and uncontrolled cupping; all 3 of these eyes had goniotomy first. The only complication was a subretinal prolene suture passage which was removed uneventfully. Results were not given stratified by which initial procedure patients had, but the necessitation of future tube shunting procedures only in patients receiving goniotomy first hints at the increased success of trabeculotomy as a more promising initial surgical procedure for pediatric GFCS. The subsequently emerging literature on the topic is largely focused on trabeculotomy for management of pediatric GFCS.

## 4. Microcatheter Trabeculotomy

A variant of the rigid probe trabeculectomy, a 360° trabeculotomy was developed in 1960, and refined in 1995, in which a 6-0 polypropylene suture was passed through the 360° circumference of Schlemm’s canal with the attempts of yielding a lower IOP than with partial opening of the lumen [59,60]. An illuminated microcatheter is used to continuously demonstrate the location of the suture in the canal to aid in correct placement of the suture and prevent placement in the suprachoroidal space [61]. Among the first reports of this procedure in GFCS eyes (aside from the one eye that received this procedure in Bothun et al., 2010) was conducted by Beck et al., which was a retrospective case series investigating success of microcatheter-guided 360° trabeculotomy in 29 eyes with various glaucomatous processes, including 4 eyes with GFCS at an average age of 5 months [53]. While this study has limited power due to low sample size of the condition at interest, and there is little information about the subgroup of GFCS patients, such as laterality, and time after initial cataract surgery, it was seen that this procedure can eliminate the need for IOP lowering therapy (2 out of 4 eyes) and can lower IOP and prevent progression of GFCS (3 of 4 eyes) without devastating complication. Bothun and Hansen also described a case of a 5 month old child with GFCS who received 300° trabeculotomy with combined pars plana capsulotomy with anterior vitrectomy for retained cortical material 4 months after cataract surgery and experienced a decrease in IOP (<20 mmHg) and had no need for further surgery [52].

To further investigate the success in microcatheter-facilitated 360° trabeculotomy in GFCS, Dao et al. conducted a retrospective chart review of 13 GFCS eyes (all aphakic) and 10 JOAG eyes [54]. Patients with anterior segment anomalies, extensive synechiae, congenital glaucoma, < 6 months of post-operative follow-up, and additional surgeries planned during study period were excluded. All surgeries were performed at two sites by one of two surgeons Out of 13 total eyes, 8 were able to receive full 360° cannulation (62%), and 1 required a second site for full cannulation. Of the remaining 5 eyes, 1 had 270° cannulation opened with the catheter and 4 had 180° rigid probe trabeculotomy. Patients were an average of 3.1 years old and mean preoperative IOP was 35.4 mmHg (±3.9) on a median of 2.5 glaucoma medications, with post-operative IOP of 21.9 mmHg (±8.6, *p* = 0.0004). Average number of IOP lowering agents was decreased from 2.8 to 1.9, but this change did not meet statistical significance. Complications included transient hypotony with choroidal effusions, recurrent scleral cyst at cutdown site, localized Descemet scar, and 2 cases of vitreous hemorrhage both requiring pars plana vitrectomy (PPV). Failure occurred in 5 eyes, only one of which had received the 180° trabeculotomy.

A larger retrospective chart review on 360° trabeculotomy in medically refractory pediatric glaucomatous eyes was subsequently conducted by Lim et al. in 2017, including 25 GFCS eyes, of which, 21 were aphakic and 4 were pseudophakic [56]. Patients with prior surgery, prior suture trabeculotomy, coexisting ocular or systemic syndromes, and insufficient follow-up time were excluded. All procedures were performed by a single surgeon at an institution where 360° trabeculotomy was standard of care for medically refractory GFCS and JOAG. All trabeculotomy procedures were performed in a standardized fashion with utilization of the iTrack microcatheter (Iscience Interventional, Menlo Park, CA, USA). If the Schlemm canal was unable to be cannulated the full 360°, either goniotomy and/or trabeculotomy with Harms trabeculotome was to be performed instead. Subconjunctival dexamethasone and cefazolin were administered, the eye was dressed in antibiotic/steroid ointment and patched. Postoperative eyes were treated with antibiotic drops, pilocarpine, and steroid drops for several weeks, and follow-up was conducted at day 1, weeks 1 and 3, and every 3–4 months as deemed medically appropriate. Success was defined as IOP ≤ 22 mm, or IOP reduction > 20% from baseline, with or without topical glaucoma medications, and without additional glaucoma surgery or devastating complications. Median patient age was 3.4 years of age, and the average length of follow-up was 31.9 months. Time since cataract surgery was not reported. Average pre-operative IOP was 31.5 (±7.5) mmHg, and patients were on an average of three drops pre-operatively. Post-operative IOP subsequently lowered to 19.7 mmHg (15.6 in eyes that achieved success, *p* < 0.001), and patients were on an average of 2.4 drops postoperatively (*p* = 0.015). No devastating post-operative complications occurred. These data suggest that microcatheter-assisted 360° trabeculotomy is a safe and effective initial surgical procedure for management of GFCS.

## 5. Degree of Schlemm Canal Manipulation and Efficacy

In performing the microcatheter-guided trabeculotomy, it is not always possible to achieve a full 360° cannulation, as has been noted in the aforementioned case studies. Anatomic abnormalities, as well as prior angle surgery or cataract surgery can lead to scarring which can deter full canalization. While prior studies on primary congenital glaucoma (PCG) have hinted that 360° canalization yields superior success rates to <180° standard trabeculotomy, these outcomes have not been thoroughly investigated in GFCS. Lim et al., 2015 conducted a retrospective case series on 360° trabeculotomy vs. <360° trabeculotomy in pediatric glaucomatous eyes, finding more significant IOP reduction in the former than the latter; however, only three eyes with GFCS were included, and specific information regarding these outcomes were not sub-stratified by disease process [55]. To determine rates of successful 360° canalization in eyes with GFCS (as well as PCG & JOAG), and to examine whether a difference in efficacy exists between varying degrees of canalization, Rojas and Bohnsack conducted a retrospective case series including 15 GFCS (12 Aphakic and 3 pseudophakic) eyes with varying degrees of suture and rigid probe trabeculotomy [58]. The average age at surgery was 7.8 years with a surgery occurring at an average of 3.4 months after initial cataract surgery, and a mean follow-up length of 3.3 years. Success was defined as IOP between 5- and 20-mm Hg without additional IOP-lowering surgery. 14 out of 15 eyes (93.3%) met this criterion, and average IOP was lowered from 27.1 mmHg (±7.0) preoperatively to 15.3 mm Hg (±3.9) Hg post-operatively (*p* < 0.001) and a decrease in number of IOP-lowering medications from 3.6 (±0.7) pre-operatively to 1.4 (±1.2) post-operatively (*p* = 0.002). Two eyes had vitreous hemorrhage requiring pars plana vitrectomy; no devastating complications were reported. Of these 15 eyes, canalization was not attempted in 4 eyes due to prior history of goniotomy surgery (2 eyes) and iridocorneal adhesions in the 180-degree opposite of the incision site (2 eyes). Of the 11 eyes with attempted 360° canalization, 4 eyes (36%) achieved 360° microcatheter canalization, 2 eyes (18%) had between 270 and 360° canalization with a combination of microcatheter and harms trabeculotomy. In 5 eyes (45%), the catheter was unable to be advanced greater than 180°, and the procedure was converted to a standard trabeculotomy with Harm’s trabectome. The only eye in the study that did not meet criteria for success was in the group which did not receive attempt at intervention, and a Baerveldt glaucoma drainage device was subsequently implanted. When compared across all glaucoma subgroups (JOAG, PCG, and GFCS), the survival rates between 360° catheter trabeculotomy, 270–360° combined trabeculotomy, and <180° trabeculotomy were statistically significant, at 5 years after surgery (100%, 92%, 69% respectively, with *p* < 0.001). These results, however, were further examined specifically in GFCS eyes, likely due to small sample size. Taken together, these results further add to the literature asserting trabeculotomy as a safe and effective procedure for management of GFCS. The higher rates of survival seen in 360° microcatheter canalization suggest that this method of trabeculotomy yields greater success in IOP control in pediatric glaucoma, but greater study is needed in GFCS eyes to determine whether this can be said to be true amongst this sub-population of pediatric glaucomatous eyes.

## 6. Two-Site Rigid Probe Trabeculotomy

Studies investigating microcatheter trabeculotomy in GFCS have largely shown success during post-operative follow-up periods with many patients not requiring additional procedures. Despite the success in IOP management seen prior, however, these procedures can potentially require additional scleral flaps or cutdowns to retrieve a misdirected or obstructed microcatheter. These additional flaps may need to be created in areas commonly utilized for tube shunts and trabeculectomy procedures later in life, which could compromise ocular structures whose integrity is imperative for future disease management. In addition, the illuminated microcatheter requires a greater cost than the standard rigid probe trabeculotomy. In a prior study by El Sayed et al. in primary congenital glaucoma, a two-site rigid-probe trabeculotomy is described in which scleral flaps are made through the superonasal and inferotemporal quadrants to gain 360° access with a rigid probe instead of a microcatheter [62]. In addition to being less costly and avoiding additional manipulation of prime ocular real estate necessary for tube and filtering procedures, outcomes were similar between the two results. In order to determine the success of two-site trabeculotomy in GFCS, El Sayed et al. performed a prospective, institutional study in Cairo, Egypt on 29 GFCS eyes (16 Aphakic, 13 pseudophakic) at an average of 5.73 years of age receiving two-site trabeculotomy with rigid probe at an average of 3.47 years after initial cataract surgery [57]. In 14 eyes, complete trabeculotomy involving all 4 quadrants was performed, and in 15 eyes, 4 quadrant canalization was not possible and only 2–3 quadrants were incised (with an average of 236 ± 44°). Average follow-up obtained was 16.9 months post-operatively. Success was defined as an IOP < 23 mmHg or 30% IOP reduction on same number or fewer topical medications without need for additional surgical procedures for IOP control, and 26 eyes (89.6%) of eyes were successful, with 15 eyes (45.5%) not requiring medications at latest follow-up. In the complete trabeculotomy group, one eye required subsequent Ahmed glaucoma valve implantation 9 months after trabeculotomy, and in the incomplete trabeculotomy group, two eyes required CPC 9 and 12 months after trabeculotomy (all aphakic eyes). Average pre-operative IOP was 26.8 mmHg (±8.2), with an average of 2.34 (±1.02) topical IOP-lowering medications. Average post-operative IOP was 14.1 mmHg (±3.1) (*p* < 0.001), with average post-operative number of drops decreased to 0.6 (±1.1, *p* < 0.001). No statistically significant difference in post-operative IOP or number of glaucoma medications needed between the complete and incomplete trabeculotomy groups. In addition, sub-analysis indicated no difference between phakic and aphakic groups in terms of IOP or medications required post-operatively. One eye experienced vitreous hemorrhage requiring pars plana vitrectomy, and another eye had progressive myopic shift requiring IOL exchange. Taken together, these results indicate that two-site trabeculotomy is a safe, efficacious, and more cost-effective method for IOP control in GFCS with decreased risk of manipulation of potential future sites of glaucoma surgery.

## 7. Discussion

Glaucoma is an insidious disease entity that remains a complication after pediatric cataract surgery [1]. Trabeculotomy in its various forms has demonstrated success in the surgical management of GFCS since as early as 1970 [12]. It is maintained as the initial surgery of choice in GFCS due to the favorable success rates demonstrated previously (with most common complication remaining vitreous hemorrhage), as well as the improved safety profile compared to traditional surgical modalities used for GFCS [12,53,54,56,63]. Many patients in the aforementioned studies have been well-controlled at the defined study follow-up without the use of IOP-lowering medication [53,56,57]. Circumferential (360° trabeculotomy) has been shown to have the highest rates of success, whether by two-site rigid probe or via microcatheter assisted suture placement [55,57,58]. These results do not appear to differ based on phakia status [57]. While circumferential trabeculotomy has repeatedly shown improved outcomes when compared to traditional <180° trabeculotomy, the data when compared to trabeculotomy between 180° and 360° in GFCS is less clear, suggesting a more complicated and dynamic relationship between degrees canalized and resulting IOP-lowering effect that would benefit from further investigation in studies with added power [57,58].

Variability is inescapable whether it be across individuals, institutions, or countries. Therefore, it becomes no surprise that there is variability seen throughout the mentioned literature in study completion and design of parameters, despite the utmost efforts to maintain systematic process. The definition of surgical success has varied across the literature from the most stringent IOP criterion presented in this review in Rojas et al. (2020), in which an IOP between 5 and 20 mmHg is detailed, as compared to that in Bothun et al. (2010) in which an IOP ≤24 mmHg is used, with the remainder of studies using definitions falling between these boundaries [51,58]. Bothun et al. (2010) references an earlier paper on diode laser CPC that utilizes an IOP cut off of 24 mmHg as a definition of success due to clinical experience seeing lack of progression of optic nerve damage with IOPs at or below this threshold, as well as the tendency of aphakic eyes with GCFS to have greater than average central corneal thickness (CCTs), and, therefore, an artificially elevated applanation pressure [37,51]. The rationale for this threshold is explicit, but because a nomogram for IOP in aphakia has yet to be developed, one may challenge the use of the particular chosen threshold. Beck et al. (2011), in addition to using an IOP threshold of <22 mmHg, also includes the condition of stable ocular dimensions and cup-to-disc ratio [53]. Dao et al. (2014) includes a condition of “clinical glaucoma stability” [54]. With variations in IOP thresholds, as well as clinical criteria used to define surgical success, combined with the, at times nebulous additional qualifiers subject to clinical judgment, one must be cautious comparing success rates amongst studies to inform surgical management.

The fruitful body of literature on the subject detailed in the aforementioned studies provides a scaffolding to which surgical management of GFCS can be anchored, and on which future studies may build.

The majority of the included literature exists as retrospective case series with small sample sizes. El Sayed et al. includes the largest sample size of GFCS eyes (29) and was the single study conducted in a prospective manner [57]. Caution must be taken when extrapolating data based on small sample sizes, as the eyes included may not always be representative of the population in ways apparent during data collection. Many of the studies have not included information about ethnicity; given the extensive study on variation in adult forms of glaucoma with ethnicity and race, the stratification of GFCS eyes by ethnicity and race may prove fruitful [64,65]. Patients with pediatric cataract surgery are at increased risk for glaucoma throughout the remainder of their lives; with the follow-up times listed on scales of mainly months, readers are given a wonderfully clear picture of GFCS shortly after angle surgery, but long-term sequelae and management have yet to be described [34]. Further investigation of the clinical question at hand would benefit from prospective studies with the use of randomization to aid in making more bolstered arguments in comparing success of various surgical treatment modalities, as well as inclusion of greater demographic data to aid in determining how similar a set of sample data is to a population at large and greater follow-up data to create a clearer picture of the entity of GFCS over the course of a lifetime.

## Figures and Tables

**Table 1:** Experimental Design & Participant Parameters in Studies Investigating Angle Surgery in Pediatric Glaucoma Following Cataract Surgery. SD = Standard Deviation; M = Male; F = Female; OS = Left Eye; OD = Right Eye. W = White, B = Black, A = Asian, AA = African American (as noted in literature), N/A = Not available.

Study	Design	Number of Surgeons	Location	Study Period	Number of GFCS Eyes Receiving Angle Surgery	Gender	Laterality	Ethnicity	Pre-Operative Lens Status	Type of Procedure	Mean Age at Surgery (SD)	Time After Cataract Surgery (SD)
Chen et al. (2004) [12]	Retrospective Chart Review	N/A	USA	1970–2002	24	N/A	N/A	N/A	24 Aphakic	Goniotomy & Rigid Probe Trabeculotomy	N/A	N/A
Bothun et al. (2010) [51]	Retrospective Chart Review	4	USA	1990–2006	14	N/A	10 OD 4 OS	N/A	N/A	Goniotomy and/or Rigid Probe Trabeculotomy (Lateral 180° Initially, repeat Nasal 180°)	Median 3 years; (range: 3 months to 9.5 years)	Median 2.9 years (range 3 months to 9 years)
Bothun & Hansen (2011) [52]	Three-Patient Case Series	1	USA	N/A	1	M	OS	N/A	Aphakic	300° Microcatheter Trabeculotomy and Anterior Vitrectomy	5 months	1 month
Beck et al. (2011) [53]	Retrospective Chart Review	N/A	Atlanta, GA, USA	July 1989–Aug. 2003	4	N/A	N/A	N/A	N/A	360° Suture Trabeculotomy	5.0 months (25th percentile: 3.0 months, 75th percentile 14.5 months)	N/A
Dao et al. (2014) [54]	Retrospective Chart Review	2	Durham, NC & Oklahoma City, OK, USA	Feb 2008–Dec 2011	13	10 M 3 F	N/A	10 W 2 B 1 A	10 Aphakic 3 Pseudophakic	360° Microcatheter Trabeculotomy	3.1 years	N/A
Lim et al. (2015) [55]	Retrospective Chart Review	N/A	Indiana, USA	2000–2012	3	N/A	N/A	N/A	N/A	360° Microcatheter Trabeculotomy	N/A	N/S
Lim et al. (2017) [56]	Retrospective Chart Review	1	USA	Feb. 2008–June 2015	25	N/A	N/A	N/A	21 Aphakic 4 Pseudophakic	360° Microcatheter Trabeculotomy	5.6 years (5.6); Median 3.4 (range 0.3–20.5 years)	N/A
El Sayed et al. (2020) [57]	Prospective, Institutional Observational Study	3	Cairo, Egypt	Jan. 2015–June 2018	29	12 M 17 F	10 OD 19 OS	29 W (Middle Eastern)	16 Aphakic 13 Pseudophakic	Two-Site Rigid Probe Trabeculotomy (180-360°)	5.73 years (1.79)	3.47 months (1.1)
Rojas et al. (2020) [58]	Retrospective Chart Review	1	Ann Arbor, MI, USA	Jan. 2013–July 2019	15	8 M 10 F	7 OD 8 OS	8 W 5 AA 2 A	12 Aphakic 3 Pseudophakic	180°–360° Microcatheter Trabeculotomy	7.8 years (5.8)	3.4 months (1.1)

**Table 2:** Pre-Operative and Post-Operative Results in Studies Investigating Angle Surgery in Pediatric Glaucoma Following Cataract Surgery. SD = Standard Deviation; IOP = Intraocular Pressure; PPV = Pars Plana Vitrectomy; ECP = Endocyclophotocoagulation; IOL = Intraocular Lens, N/A = Not available.

Study	Mean Pre-Operative IOP in mmHg (SD)	Mean Pre-Operative Number of Topical IOP-Lowering Agents (SD)	Average Length of Follow-up (SD)	Mean IOP at Latest Post-Operative Follow-Up Visit (SD)	Mean Number of Topical IOP-Lowering Agents at latest Post-Operative Follow-Up Visit (SD)	IOP Reduction *p*-Value	Topical IOP Lowering Agent Number Reduction *p*-Value	Definition of Success	Number of Eyes Achieving Success	Number of Eyes Achieving IOP Control Without Topical Therapy	Complications Requiring Intervention	Time After Cataract Surgery (SD)
Chen et al. (2004) [12]	N/A	N/A	N/A	N/A	N/A	N/A	N/A	IOP ≤ 21 mmHg with or without medications and no need for further surgery	16(16%)	N/A	N/A	N/A
Bothun et al. (2010) [51]	35 mmHg (10)	N/A	4.7 years	22 mmHg (4)	N/A	*p* = 0.0005	N/A	IOP ≤ 24 mmHg with or without topical medication, lack of sight-threatening complication, avoidance of trabeculectomy or tube shunt	8 (57.1%)	N/A	Subretinal prolene suture passing, removed uneventfully	None
Bothun & Hansen (2011) [52]	N/A	N/A	Case1: 18 months Case2: 18 months Case 3: 7 months	Case1: <20 mmHg Case2: <25 mmHg Case 3: <22 mmHg	Case1: dorzolamide and timolol Case2:0 Case3: 0	N/A	N/A	Case1: IOP < 20 mmHg Case2: IOP < 25 mmHg Case 3: IOP < 22 mmHg	Cases 1,2 and 3: 3 (100%)	2 (66.7%)	Cases 1,2 and 3: The patient recovered without complications or further surgery.	N/A
Beck et al. (2011) [53]	33.0 mmHg (7.2)	N/A	1.6 years (25th percentile 0.3, 75th percentile 3.7)	21.8 mmHg (5.3)	N/A	N/A	N/A	IOP < 22 mmHg with allowance for maximum medical therapy with stable cup-to-disc ratio (within 0.1 of pre-operative value) and no further surgery	3 (75%)	2 (50%)	N/A	N/A
Dao et al. (2014) [54]	35.4 mmHg (4.7) [35.5 mmHg (3.9) in successful eyes]	Median 2.5 [2.8 in successful eyes]	Median 30 months (range 6 to 47)	21.9 mmHg (8.6) [15.4 (3.3) in successful eyes]	1.9 (in successful eyes)	*p* = 0.0004 [*p* < 0.0001 in successful eyes]	*p* = 0.07	IOP < 22 mmHg with > 30% reduction without disease progression, oral glaucoma medication, or additional glaucoma surgery	8 (62%)	N/A	Vitreous Hemorrhage in 2 eyes requiring PPV	Transient Choroidal Effusion in setting of Hypotony
Lim et al. (2015) [55]	Traditional group, 28.75 mmHg (8.80); 360-degree group, 30.35 mmHg (6.04).	Traditional group, 0.87 (1.03); 360-degree group, 1.07 (0.83).	1 Year	Traditional group, 17.05 mmHg (5.92); 360-degree group, 11.0 mmHg (2.31).	N/A	all *p* < 0.01	N/A	Surgical failure was defined as the need for additional glaucoma procedure(s) after initial trabeculotomy	Traditional group, 45 (58.44%); 360-degree group, 12 (85.71%).	N/A	N/A	N/A
Lim et al. (2017) [56]	31.5 mmHg (7.5)	3.0 (1)	31.9 months (26.1)	19.7 mmHg (7.7) [15.6 (3.9) in successful eyes]	2.4 (1.1)	*p* < 0.001	*p* = 0.015	IOP ≤ 22 mmHg or IOP reduction > 20% from baseline, with or without topical glaucoma medications, considered adequate for glaucoma severity without additional glaucoma surgery or devastating complication	18 (72%)	2	Vitreous Hemorrhage in 2 eyes requiring PPV, 1 of which also required ECP	None
El Sayed et al. (2020) [57]	26.8 mmHg (8.2)	2.34 (1.02)	16.9 months	14.1 mmHg (3.1)	0.6 (1.1)	*p* < 0.001	*p* < 0.001	IOP < 23 mmHg or 30% IOP reduction on same number or fewer topical medications without need for additional procedure	26 (89.6%)	15 (45.5%)	Vitreous hemorrhage requiring PPV; progressive myopic shift requiring IOL exchange	None
Rojas et al. (2020) [58]	27.1 mmHg (7.0)	3.6 (0.7)	3.3 years (2.4)	15.3 mmHg (3.9)	1.4 (1.2)	*p* < 0.001	*p* = 0.002	IOP 5–20 mmHg, no additional IOP-lowering surgery	14 (93.3%)	N/A	Vitreous Hemorrhage Requiring PPV	None
